# Erratum zu: COVID-19 – neue Herausforderungen in der Dysphagie- und Atemtherapie

**DOI:** 10.1007/s00115-022-01365-4

**Published:** 2022-08-11

**Authors:** Ulrike Frank, Katrin Frank

**Affiliations:** 1 Department Linguistik, Swallowing Research Lab, Universität Potsdam, Karl-Liebknecht-Str. 24–25, 14.202, 14476 Potsdam, Deutschland; 2 St. Vincenz Krankenhaus, Paderborn, Deutschland

**Erratum zu:**

**Nervenarzt 2021**

10.1007/s00115-021-01162-5

Der Originalbeitrag wurde korrigiert.

In diesem Artikel fehlte die Lizenzangabe. Sie hätte „Open Access. Dieser Artikel wird unter der Creative Commons Namensnennung 4.0 International Lizenz veröffentlicht, welche die Nutzung, Vervielfältigung, Bearbeitung, Verbreitung und Wiedergabe in jeglichem Medium und Format erlaubt, sofern Sie den/die ursprünglichen Autor(en) und die Quelle ordnungsgemäß nennen, einen Link zur Creative Commons Lizenz beifügen und angeben, ob Änderungen vorgenommen wurden. Die in diesem Artikel enthaltenen Bilder und sonstiges Drittmaterial unterliegen ebenfalls der genannten Creative Commons Lizenz, sofern sich aus der Abbildungslegende nichts anderes ergibt. Sofern das betreffende Material nicht unter der genannten Creative Commons Lizenz steht und die betreffende Handlung nicht nach gesetzlichen Vorschriften erlaubt ist, ist für die oben aufgeführten Weiterverwendungen des Materials die Einwilligung des jeweiligen Rechteinhabers einzuholen. Weitere Details zur Lizenz entnehmen Sie bitte der Lizenzinformation auf http://creativecommons.org/licenses/by/4.0/deed.de.“ lauten sollen.

